# Grandchild Caring and Late-Life Depression: A Comparative Longitudinal Study in China and Europe

**DOI:** 10.1093/geroni/igab046.1519

**Published:** 2021-12-17

**Authors:** Yazhen Yang

**Affiliations:** University of Southampton, Southampton, England, United Kingdom

## Abstract

The impact of grandparenting on the grandparents’ health has been relatively under-studied, and international comparisons can provide useful lessons for grandparents and policymakers. This study examined country differences in the effects of grandchild care provision on the grandparents’ depression in Italy, Spain, China, Denmark and Sweden using the longitudinal Harmonised CHARLS and SHARE data collected between 2010-5. Controlling for the grandparents’ depression in 2011, grandparents providing non-intensive grandparental care in China, Sweden and Denmark in 2013 were less likely to report depression in 2015 compared to those who did not provide any care in 2013. Such effects were more pronounced among grandmothers compared to grandfathers. The results indicate that the effects of grandchild caring on the grandparents’ depression in China was comparable to Denmark and Sweden. Future research can focus on identifying the causal pathways between grandparenting and wellbeing, and the implications of such pathways for older persons’ wellbeing worldwide.

